# Are Platelet-Related Parameters Prognostic Predictors of Renal and Cardiovascular Outcomes in IgA Nephropathy?

**DOI:** 10.3390/jcm13040991

**Published:** 2024-02-08

**Authors:** Balázs Sági, Tibor Vas, Botond Csiky, Judit Nagy, Tibor József Kovács

**Affiliations:** 1 2nd Department of Internal Medicine and Nephrology, Diabetes Center, Clinical Center, Medical School, University of Pécs, 7624 Pécs, Hungary; balazs.sagidr28@gmail.com (B.S.); botond.csiky@gmail.com (B.C.); judit.nagy@pte.aok.hu (J.N.); 2 Triton Life Dialysis Center, 7624 Pécs, Hungary

**Keywords:** platelet-to-albumin ratio, platelet-to-lymphocyte ratio, chronic kidney disease, IgA nephropathy, renal and cardiovascular prognosis

## Abstract

**Background:** IgA nephropathy (IgAN) is associated with chronic inflammation. Platelet-related parameters, such as the platelet (PLT) count, platelet-to-albumin ratio (PAR), and platelet-to-lymphocyte ratio (PLR), were examined as potential prognostic indicators for renal and cardiovascular (CV) outcomes in IgAN. We were interested in whether platelet-related parameters are risk factors for ESKD and CV events in IgAN patients. **Methods:** In a monocentric retrospective study, 124 IgAN patients were divided into two groups based on the cut-off value of the PAR. All-cause mortality, major CV events, and end-stage renal disease were the primary combined endpoints. Secondary endpoints, such as CV or renal endpoints, were also analyzed separately. **Results:** The patients’ mean age was 43.7 ± 13.5 years, and the follow-up time was 124 ± 67 months. The K-M curve showed that the PLR, PAR, and PLT were strongly associated with primary combined (*p* = 0.002, *p* = 0.004, *p* = 0.001) and renal outcomes (*p* < 0.001, *p* < 0.001, *p* < 0.001), but not with CV outcomes in IgAN. However, when combined with left ventricular hypertrophy (LVH) or metabolic syndrome (MetS), the PAR was found to be a significant predictor of both primary (*p* < 0.001, *p* < 0.001) and secondary outcomes (*p* = 0.001 and *p* = 0.038; *p* = 0.001 and *p* = 0.015). Additionally, the PLR correlated with albuminuria (r = −0.165, *p* = 0.033) and LVH (r = −0.178, *p* = 0.025), while PLT correlated with eGFR (r = 0.158, *p* = 0.040). **Conclusions.** Elevated PARs and PLRs may predict progression to end-stage kidney disease, but in combination with LVH and MetS, they were related to CV events in IgAN. The determination of PARs and PLRs can be useful and cost-effective parameters for assessing both cardiovascular and renal risks in IgAN.

## 1. Introduction

Immunoglobulin A nephropathy (IgAN) is one of the most common types of primary glomerulonephritis worldwide [1]. Based on the immune pathomechanism of IgAN, it is also associated with chronic inflammation. Studies showed that about 10–30% of IgAN patients may lose their kidney function gradually and progress to end-stage kidney disease (ESKD) within 10 years from the time of diagnosis, costing considerable socioeconomic resources [2,3]. The identification of patients with IgAN who are at a high risk of a progressive reduction in renal function is worthwhile, but the patients die from cardiovascular (CV) causes before reaching ESKD. As a result, it is crucial to recognize high-risk patients and take appropriate action. Accumulating evidence has illustrated that IgAN is an immune system disease where the activation of inflammation is closely related to the outcome of the disease [4,5]. Platelet-related parameters, including platelets (PLTs), the platelet-to-albumin ratio (PAR), and the platelet-to-lymphocyte ratio (PLR), are easy to obtain clinically and have been proven to be novel prognostic indicators for several different inflammatory diseases [6,7,8,9,10], such as rheumatoid arthritis, spondylarthritis, psoriasis, head and neck cancers, and soft tissue sarcoma. Moreover, the results of multiple studies consistently report that the PAR and PLR are associated with inflammation and have been described as emerging inflammation indexes [6,7,8,9,10]. In addition to hemostasis, platelets may also be able to trigger and aggravate inflammation by interacting with immune cells and secreting proinflammatory cytokines [11]. Nevertheless, few studies have demonstrated the precise relationship between platelet-related parameters and IgAN. A recent study by Tan et al. found that the PAR could be an independent renal risk predictor in a Chinese ethnic population [12]. Accordingly, we examined the data of 124 predominantly male patients with IgAN to determine whether platelet-related parameters are risk factors for ESKD and CV events in IgAN patients.

## 2. Materials and Methods

### 2.1. Selection of Patients

This monocentric retrospective study included 150 patients with IgAN diagnosed by renal biopsy at the 2nd Department of the Internal Medicine, Nephrology, and Diabetes Center of the University of Pécs Clinical Center between January 2003 and December 2018. Among these 150 patients, only one withdrew their informed consent. Twelve individuals whose data were missing during follow-up and 13 subjects treated with immunosuppression were excluded from the study. Ultimately, 124 patients were enrolled in our study. All patients were monitored regularly at the outpatient clinic for at least one to three months following the kidney biopsy. This study was approved by the Ethics Committee of the University of Pécs (3170/2008), and all methods were carried out according to relevant guidelines and regulations. All patients completed the written informed consent form to be included in this study.

### 2.2. Clinical and Histological Data Collection

At the time of the renal biopsy and the follow-up visit, demographic information and clinical data were gathered. Hypertension was defined as SBP > 140 mmHg and/or DBP > 90 mmHg at rest. The Oxford classification (M0/M1, mesangial hypercellularity; E0/E1, endocapillary hypercellularity; S0/S1, glomerulosclerosis; T0/T1/T2, tubular atrophy and interstitial fibrosis; and C, cellular or fibro-cellular crescents) was used to evaluate the pathological lesions [5].

### 2.3. Renal and Cardiovascular Endpoints

The primary composite endpoint included cardiovascular (CV) outcomes (containing overall mortality), coronary intervention (acute coronary events (ACSs)), and stroke, as well as renal outcomes (ESKD development: renal replacement therapy was started or eGFR < 15 mL/min/1.73 m^2^). Subsequently, CV and renal endpoints were analyzed separately as secondary endpoints.

### 2.4. The Definition of Platelet-Related Parameters

PLTs (G/L) were measured as the absolute platelet count of a routine blood examination, where platelets were measured per microliter of blood. The PAR (G/g) is the absolute number of platelets divided by serum albumin. The PLR is the ratio of platelets to lymphocytes. In this study, the cut-off was defined as PAR, PLR, and PLT (maximum Youden index: sensitivity + specificity − 1). Routine laboratory examinations (hemoglobin, uric acid, total cholesterol, triglyceride, and HDL cholesterol) and urine albumin measurements were also performed.

### 2.5. Statistical Analysis

Univariate and multivariate linear regression were used to identify independent predictor variables or risk factors. Cox regression was used to compare parameters and renal survival. Normally, continuous variables are expressed as the means ± SD and compared using a *t*-test. *p* < 0.05 was considered statistically significant. Nonparametric variables are usually expressed as medians with interquartile ranges and were compared using either the Mann–Whitney U or the Kruskal–Wallis test. Categorical variables were compared using an χ^2^ test. The data were analyzed using Microsoft Excel 2021 (version 16) and the SPSS 29.0.1.1 software.

## 3. Results

**Figure 1:**
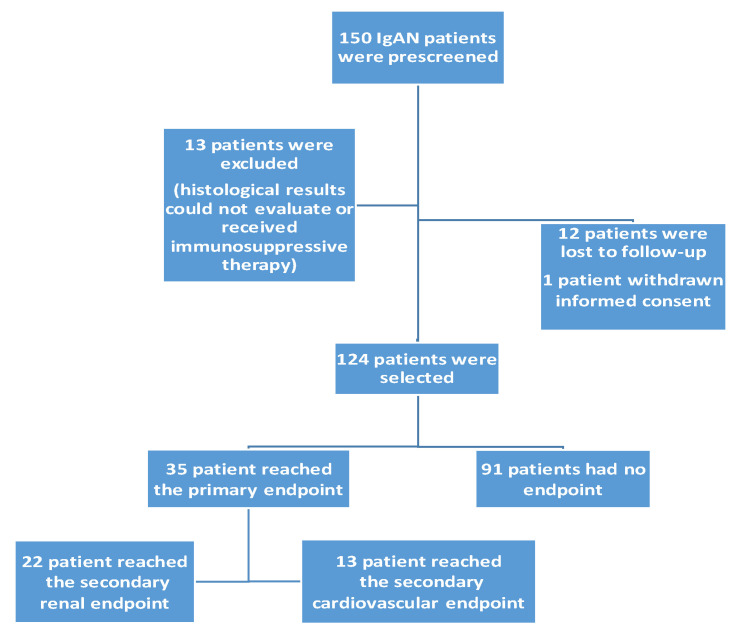
The flow chart of recruited patients.

We included 124 patients with IgAN, who were followed for an average of 124 ± 67 months between 2005 and 2021. Figure 1 shows a flow chart of the recruited patients. The mean age of the patients was 43.7 ± 13.5 years, of whom 74 were male. The majority of patients (84%) were hypertensive, and 27% were diabetic at the time of the IgAN diagnosis. The main clinical data and the incidence of risk factors are detailed in Table 1.

In our study, 35 (28%) of the 124 IgAN patients reached the primary combined endpoint, 22 (18%) the secondary renal endpoint, and 13 (10%) patients reached the secondary cardiovascular endpoint.

The mean PLT, PLR, and PAR of the 124 patients were 140.14 ± 65.18 (G/L), 5.78 ± 1.89, and 238.9 ± 68.88 (G/g). The patients were divided into two groups according to the PAR AUC values determined by an ROC analysis. The clinical data of patients divided into two groups based on a low and high PAR is listed in Table 1. These two groups of IgAN patients differed significantly in gender, mean blood pressure, LVH, and diastolic dysfunction. There were no differences between the two groups regarding age, eGFR, metabolic parameters (dyslipidemia, diabetes (DM), obesity, body mass index (BMI), hypertension), ejection fraction, laboratory results (hemoglobin (Hb), albuminuria (AU), uric acid (UA), using angiotensin-converting enzyme inhibitor (ACEI), angiotensin II receptor blocker (ARB), statins, or MEST-C score.

The patients were divided into two groups according to whether they reached the primary endpoint or not. Clinical data of the patients was divided into two groups based on the presence or absence of the primary combined endpoint, listed in Table 1. These two groups of IgAN patients differed significantly in gender, age, mean blood pressure, systolic diurnal index, ejection fraction, hypertension, BMI, DM eGFR, smoking, metabolic syndrome occurrence, PLR, PAR, AU, UA, and LVH. In the group with a combined primary endpoint, there was significantly higher usage of antihypertensive drugs such as ACE/ARB, BB, CCB, and statins. There were no differences between the two groups regarding pulse pressure, dyslipidemia, duration of kidney disease, or laboratory results (platelet count, Hb, total cholesterol, HDL cholesterol, and triglyceride). 

**Figure 2:**
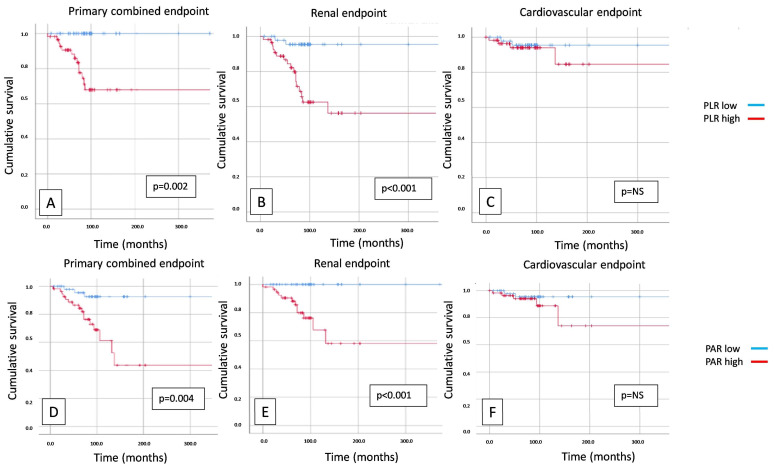

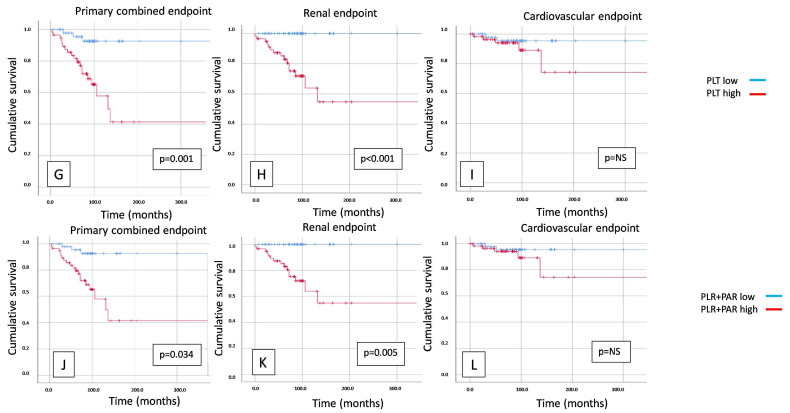
Kaplan–Meier curves show primary combined (**A**), renal (**B**), and cardiovascular (**C**) endpoints in the case of platelet-to-lymphocyte ratio (PLR); primary combined (**D**), renal (**E**), and cardiovascular (**F**) endpoints in case of platelet-to-albumin ratio (PAR); primary combined (**G**), renal (**H**), and cardiovascular (**I**) endpoints in case of platelet count (PLT); primary combined (**J**), renal (**K**), and cardiovascular (**L**) endpoints in case of PLR and PAR. NS: not significant.

In our study, 35 (28%) of the 124 IgAN patients reached the primary combined endpoint, 22 (18%) reached the secondary renal endpoint, and 13 (10%) reached the secondary cardiovascular endpoint. A survival analysis showed significant differences between high and low PLRs in the case of the primary combined endpoint (*p* = 0.002) (Figure 2A) and the secondary renal endpoint (*p* < 0.001) (Figure 2B), but the secondary cardiovascular endpoint was not significant (*p* = NS) (Figure 2C). There was also a significant difference between high and low PARs in the case of the primary combined endpoint (*p* = 0.004) (Figure 2D) and the secondary renal endpoint (*p* < 0.001) (Figure 2E), but in this case, the secondary cardiovascular endpoint was not significant (Figure 2F). The high PLTs compared to the low PLTs showed significantly worse survival in the case of the primary combined endpoint (*p* = 0.001) (Figure 2G) and the secondary renal endpoint (*p* < 0.001) (Figure 2H), but the cardiovascular endpoint was not significant (Figure 2I). In that case, if we combined the high PARs with the high PLRs and compared them with the low PAR and PLR group, we obtained the same results: the primary combined endpoint and the secondary renal endpoint showed significantly worse survival (*p* = 0.034 and *p* = 0.005) (Figure 2J–K) in the high PAR and PLR group, but there was no significant difference between the two groups in terms of cardiovascular outcomes (Figure 2L).

The PAR was correlated with gender and segmental glomerulosclerosis in histology (S). The PLR was correlated with albuminuria and left ventricular hypertrophy; PLTs were correlated with gender and eGFR (Table 2). There were no significant differences between male and female patients’ primary and secondary renal and cardiovascular outcomes. There was no significant correlation between the PAR, PLR, and PLT and the drugs (ACEI/ARB, beta-blockers (BBs), calcium channel blockers (CCBs), and statins) taken by the patients.

When we analyzed 14 parameters (gender, age, dyslipidemia, obesity, hypertension, diabetes, eGFR, albuminuria, separate MEST-C histological characteristics, and left ventricular hypertrophy (LVH)) by uni- and multivariate linear regression analysis, only gender was a significant influencing factor for the PAR; LVH for PLR; and gender and hypertension for PLT (Table 3).

The PLR, gender, dyslipidemia, and albuminuria were associated with the primary combined endpoints; left ventricular hypertrophy and albuminuria with the secondary renal endpoints; and gender, age, and diabetes with the cardiovascular endpoints by multivariate Cox regression (Table 4).

**Figure 3:**
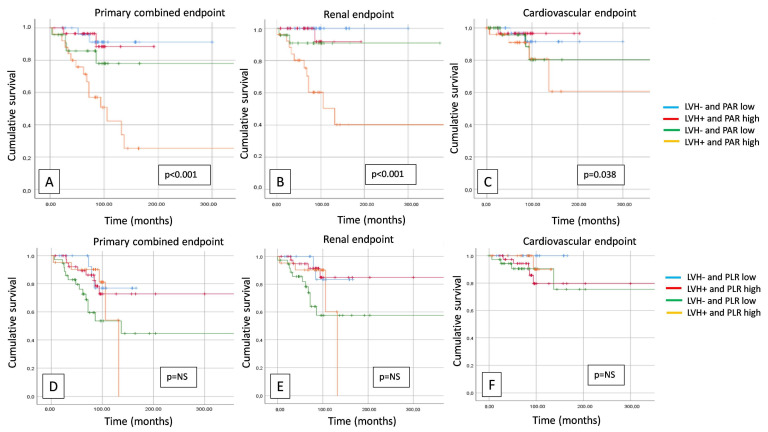
Kaplan–Meier curves show primary combined (**A**), renal (**B**), and cardiovascular (**C**) endpoints in case of left ventricular hypertrophy (LVH) (positive/negative) and platelet-to-albumin ratio (PAR) (high/low), and primary combined (**D**), renal (**E**), and cardiovascular (**F**) endpoints in case of left ventricular hypertrophy (LVH) (positive/negative) and platelet-to-lymphocyte ratio (PLR) (high/low). NS: not significant.

**Figure 4:**
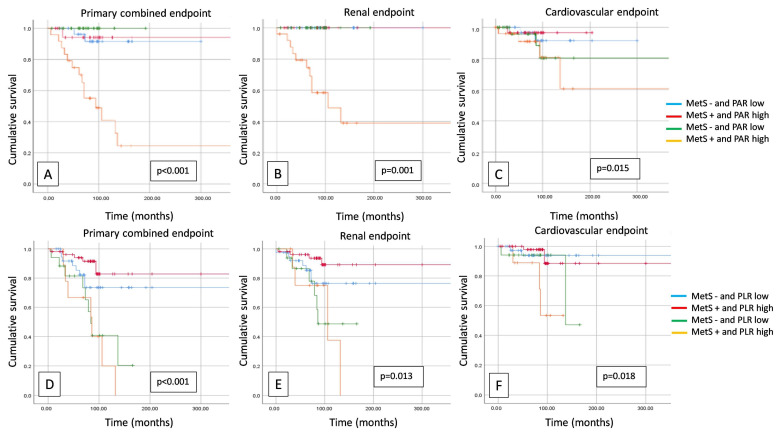
Kaplan–Meier curves show primary combined (**A**), renal (**B**), and cardiovascular (**C**) endpoints in case of metabolic syndrome (MetS) (positive/negative) and platelet-to-albumin ratio (PAR) (high/low), and primary combined (**D**), renal (**E**), and cardiovascular (**F**) endpoints in case of metabolic syndrome (MetS) (positive/negative) and platelet-to-lymphocyte ratio (PLR) (high/low).

In our follow-up study, the presence of a high PAR combined with the presence of LVH significantly worsened survival in the cases of primary endpoints (*p* < 0.001), renal endpoints (*p* < 0.001), and also in CV endpoints (*p* = 0.038) (Figure 3), but there was no significant difference in the case of the combination of PLR and LVH in neither endpoint. The presence of metabolic syndrome (MetS) combined with a high PAR and high PLR significantly impaired survival at both primary (*p* < 0.001; *p* < 0.001) and secondary renal (*p* = 0.001, *p* = 0.013) and cardiovascular endpoints (*p* = 0.015; *p* = 0.018) (Figure 4).

## 4. Discussion

IgAN is a frequently occurring disease that has a variety of factors contributing to its development and progression [1]. Most researchers agree that autoimmune (forming IgG/galactose-deficient IgA1 antibodies = antiGd-IgA1-ab) and inflammation (activation of the complement cascade) processes play significant roles in the pathomechanism and progression of IgAN, even though the exact pathophysiology of the disease is still unknown [1,2,3].

In addition to their traditional role in blood clotting, platelets (PLTs) also play an important role in immunity, according to recent studies [13,14,15]. There is evidence that platelets release pro-inflammatory mediators, such as cytokines and chemokines, which influence the inflammatory processes [16,17]. Therefore, it is not surprising that in many diseases with immune pathomechanisms, the study of platelet counts has come to the fore.

According to recent studies, chronic kidney disease (CKD), diabetic nephropathy, focal segmental glomerulosclerosis, and several other renal disorders’ outcomes are associated with platelet-related factors [18,19,20,21,22]. As a result, it is assumed that platelet numbers may reflect the activity of the immune response during IgAN, which was demonstrated by our results.

The significant role of lymphocytes in immunity is much better known. Therefore, the platelet/lymphocyte ratio (PLR) can also characterize inflammation in various diseases, which may be related to disease outcomes.

Serum albumin is thought to be linked to inflammation in addition to reflecting the body’s nutritional state [23,24]. A new indicator—the platelet-to-albumin ratio (PAR)— that combines the two (PLT and albumin) is suggested as having the potential to more accurately reflect the body’s inflammatory state [25].

In a previous study, the PAR was correlated with one of the most commonly used inflammatory serum markers, such as C-reactive protein (CRP); however, CRP and interleukin-6 (IL-6) are not routine examination items for patients with IgA nephropathy, and there is no guideline recommending routine examinations of CRP and IL-6 in IgAN patients. Unfortunately, we did not examine the association between CRP, IL-6, and PAR [26,27].

Several studies have shown that the PAR is indeed an inflammatory index [28]. As a result, the PAR was thought to be a preferred marker due to its low cost and ease of calculation in the clinic. Therefore, the PAR, a new inflammatory marker, may be used in clinical practice. In addition, the PAR is a combination marker that seems to be more accurate in risk prediction than other platelet-related parameters. It has been reported that the PAR might be more stable and less likely to be affected by dynamic physiological conditions than other platelet parameters and/or inflammatory markers [29,30].

According to Huang et al. [7], there was a favorable correlation between the PAR and the disease activity of axial spondylarthritis. The use of the PAR to forecast patient mortality in cases of severe fever and thrombocytopenia syndrome was also demonstrated [31]. Similar to how peritoneal dialysis patients’ results are predicted by the PAR [32], the PAR may therefore evolve into a novel prognostic indicator in the future, according to the consistent findings of several articles [9,10] in diseases where the presence of inflammation is much more obvious.

Surprisingly, the PAR could still forecast a poor prognosis for IgAN in which the known signs of inflammation are not so pronounced. Recent research has shown that various chronic inflammatory indicators and platelet counts are positively correlated in end-stage kidney disease and peritoneal dialyzed patients [33,34].

IgAN’s pathophysiology and progression are probably influenced by an abnormal immune response by forming abnormal galactose-deficient IgA1, the process of which can be characterized as chronic inflammation and the activation of various immune mediators [35].

Tan et al. also found in IgAN patients that the PAR seemed to be a better marker of adverse renal outcomes than the PLR, albumin, and PLTs, suggesting that the PAR was the only platelet-related parameter that could be used as an independent risk factor [12].

In our study, the K-M curve revealed that PLTs, the PLR, and the PAR were all significantly linked with the renal but not the cardiovascular outcomes of IgAN, indicating that each of them may be used as prospective predictors of renal endpoints, as Tan et al. [12] reported in their cohort. In our previous studies, we found that the left ventricular mass index (LVMI) and the development of metabolic syndrome (MetS) in IgAN patients have an impact on cardiovascular outcomes [36,37]. Inflammatory parameters may indicate further factors that are responsible for the accelerated manifestation of the endpoints. It is supported by the stronger correlation of the outcome if we were to take into consideration the presence of MetS as well. Although the PAR and PLTs were correlated with gender, there were no significant differences between male and female patients’ primary and secondary outcomes.

The deposition of IgA in the glomeruli may start with the unspecific activation of vascular mediators, which will lead to BP elevation and glomerulosclerosis [12]. This phenomenon may be unique to all chronic glomerular diseases where patients have hypertension-induced LVH and deteriorating renal function, and they also have metabolic syndrome. Our previous data confirmed that these two cardiometabolic alterations cause higher cardiovascular morbidity and mortality [36,37]. So, it is indispensable to recognize the high CV risk of IgAN patients and treat them more aggressively to reduce their risk. That is why we combined the PAR and the PLR with cardiometabolic factors, which reinforced each other. The PAR may be more predictive of prognosis and outcomes in patients who are already at higher CKD stages at the time of biopsy, which may be because mild renal impairment patients progress to ESRD relatively slowly, while patients with severe renal impairment develop to the endpoint somewhat quickly [38].

Tan et al. [12] found a significant difference between low and high PARs in endocapillary hypercellularity (E0/E1) in their cohort of 966 IgAN patients. This indicates that the severity of the histological abnormality is only one factor in the progression of the disease. This may draw attention to the fact that, in some cases (a high PAR), immunosuppressive treatment has a right to exist. In our study, there was no significant difference in the histological classification (Oxford) between the high and low groups of PARs, which may be explained by different populations (Chinese vs. Caucasians) or lower case numbers.

In Yi et al.’s study, crescent formation in the glomeruli and the platelet count were also risk predictors of poor prognosis in IgAN [39], but the platelet count was not an independent predictor of crescent formation in IgAN. We also found no correlation between these two parameters in our study. However, we found a weak but significant correlation between PAR and segmental glomerulosclerosis (S0/S1), which could draw attention to the early risk stratification and treatment of high-risk patients.

Previous studies have suggested that proteinuria is a well-established risk factor for kidney function decline in IgAN [40]. We also demonstrated that proteinuria was shown to be an independent risk factor for the PLR, but not for the PAR or PLTs. In certain diseases, the PAR shows a closer correlation, while in other diseases, the PLR shows a closer correlation with proteinuria, which may indicate activity differences at different levels of inflammatory processes and different degrees of activation in the complement cascade, which can be caused by different changes in PLTs, lymphocytes, and albumin values.

Gan and Zeng et al. showed in their prospective cohort and meta-analysis that CKD patients have higher PLR values compared to non-CKD patients, and PLR values were highly associated with all-cause mortality in CKD patients. The PLR is a valid predictor as a clinically accessible indicator for patients with CKD [41,42]. Our study found associations between the PLR and combined and renal endpoints, but not with the cardiovascular endpoints, which supports this observation in smaller but more uniform kidney disease.

Chen et al. demonstrated in their study that the PLR was independently associated with CV events in a small number of peritoneal dialysis patients during a 22-month median follow-up period [43]. In these patients, peritoneal inflammatory activation was consistently present and should be much higher (more active) than in IgAN.

In our study, we could not confirm this, but the difference could have been that the peritoneal dialysis patients were in severe CV status; in our patients, they were not dialyzed and had better renal function and a better CV status. If we combined the PAR and PLR with the presence of LVH and MetS, strengthening the results of our study, the CV endpoints also became significant. Metabolic alterations like MetS cause the further acceleration of atherosclerosis and the progression of kidney alterations. Therefore, we could recommend cardiovascular and metabolic screening at least annually for high-risk, asymptomatic (especially with a high PAR or PLR) IgAN patients.

### Limitations of this Study

First, this is a one-center study with limited follow-up time. Second, the cut-off value is based on this cohort. Therefore, it may not be appropriate for other populations and races. Third, since this was a retrospective study, there were no detailed data for regular follow-ups in each subject, so a dynamic analysis of the PAR could not be performed. Fourth, retrospective analyses are prone to residual confounding effects of comorbidity and medications that affect blood cell counts and selection bias. This is why prospective studies need to be conducted with large sample sizes. Fifth, this study included more men than women, which may bias the results.

## 5. Conclusions

Increased PARs and PLRs may predict the progression to end-stage renal disease, but combined with LVH and MetS, they were related to CV events in IgAN. Determining the PAR and PLR, which are simple and cheap parameters, may be useful parameters not only for CV risk but also for the stratification of renal risk in IgAN.

## Figures and Tables

**Table 1:** Baseline characteristics.

Clinical Data	IgAN Patients (*n* = 124)	PAR High (*n* = 61)	PAR Low (*n* = 63)	*p*	Without a Combined Endpoint (*n* = 91)	With a Combined Endpoint (*n* = 33)	*p*
Man/woman (*n*/%)	83/29 (74/26)	48/13 (79/21)	35/28 (55/45)	0.004	53/38 (58/42)	28/58(85/15)	<0.001
Age (year)	43.7 ± 13.5	43.6 ± 11.7	43.9 ± 11.2	NS	40.7 ± 12.3	53.1 ± 10.0	<0.001
Average systolic/diastolic RR (mmHg)	124/74 ± 14/9	127/75 ± 15/9	120/73 ± 11/8	0.003	121/72 ± 13/8	129/77 ± 14/8	0.003
24 h pulse pressure (mmHg)	49.6 ± 10.7	52.2 ± 12.8	47.1 ± 7.7	0.012	48.9 ± 8.0	51.8 ± 12.5	NS
Diurnal index systolic (%)	9.66 ± 5.6	10.2 ± 6.2	9.2 ± 5.2	NS	10.4 ± 5.9	7.6 ± 7.8	0.033
Metabolic parameters							
Hypertension (*n*, %)	94 (84)	51 (81)	43 (70)	NS	62 (68)	30 (91)	<0.001
BMI (kg/m^2^)	26.6 ± 4.6	26.7 ± 4.5	26.5 ± 4.7	NS	26.2 ± 4.4	27.8 ± 4.7	0.048
Dyslipidemia (*n*, %)	58 (52)	32 (51)	26 (43)	NS	38 (42)	18 (54)	NS
Diabetes (*n*, %)	30 (27)	15 (24)	15 (24)	NS	14 (15)	16 (48)	0.001
eGFR (mL/min/1.73 m^2^)	84.5 ± 32.4	83.8 ± 29.6	85.2 ± 27.8	NS	93.0 ± 33.5	62.1 ± 30.7	0.001
Duration of kidney disease (year)	10.8 ± 9.4	11.5 ± 10	10 ± 9	NS	9.5 ± 9.3	10.2 ± 10.8	NS
Smoking (*n*, %)	21 (19)	11 (17)	10 (16)	NS	11 (12)	9 (27)	0.012
Metabolic syndrome (*n*, %)	27 (24)	14 (22)	13 (21)	NS	11 (12)	16 (48)	<0.001
Platelet-related parameters							
PLR	140.14 ± 65.18	158.05 ± 73.05	122.23 ± 50.15	0.001	132.67 ± 35.88	155.58 ± 84.44	0.037
PAR (G/g)	5.78 ± 1.89	7.12 ± 1.64	4.41 ± 0.89	<0.001	5.43 ± 1.84	6.03 ± 1.71	0.039
PLT (G/L)	238.9 ± 68.88	290 ± 51.29	187.7 ± 40.24	<0.001	244.27 ± 64.86	221.35 ± 69.48	NS
Echocardiographic parameters							
LVEF (%)	62.4 ± 6.5	62.9 ± 7.7	62.5 ± 4.9	NS	63.4 ± 6.3	61.0 ± 6.1	0.037
LVMI (g/m^2^)	107.7 ± 22.8	110.5 ± 23.2	104.9 ± 16.1	0.034	99.7 ± 19.7	127.1 ± 17.1	<0.001
LVM (g)	204.4 ± 51.4	239.0 ± 48.8	194.9 ± 44.0	0.028	199.1 ± 46.5	241.2 ± 48.7	<0.001
LVEDD (cm)	5.09 ± 0.4	4.93 ± 0.39	5.05 ± 0.41	NS	5.88 ± 0.42	5.14 ± 0.33	NS
DD (*n*/%)	37 (47)	24 (39)	13 (21)	0.025	33 (36)	24 (72)	0.001
Pathological lesions							
M (0/1) (*n*/%)	52 (46)	29 (46)	23 (38)	NS	34 (37)	16 (48)	NS
E (0/1) (*n*/%)	2 (2)	1 (1.6)	1 (1.6)	NS	1 (1)	0 (0)	NS
S (0/1) (*n*/%)	22 (20)	14 (22)	8 (13)	NS	14 (15)	6 (18)	NS
T (0/1/2) (*n*/%)	56 (50)	27 (43)	29 (47)	NS	27 (29)	29 (88)	<0.001
C (0/1) (*n*/%)	28 (25)	17 (27)	11 (18)	NS	18 (20)	10 (30)	NS
Laboratory results							
Hb (g/dL)	13.6 ± 1.53	13.6 ± 1.54	13.7 ± 1.56	NS	13.9±	13.3±	NS
AU (mg/L)	484.6 ± 658.4	494.8 ± 521.8	431.4 ± 550.9	NS	361.2±	731.7±	0.002
UA (umol/L)	320.5 ± 76.7	318.1 ± 68.8	342.3 ± 76.7	NS	318.4±	363.4±	0.015
Total cholesterol (mmol/L)	5.03 ± 1.21	4.95 ± 1.41	4.98 ± 0.95	NS	5.39±	5.19±	NS
HDL cholesterol (mmol/L)	1.28 ± 0.51	1.23 ± 0.36	1.31 ± 0.64	NS	1.34±	1.32±	NS
TG (mmol/L)	1.69 ± 1.05	1.76 ± 1.12	1.71 ± 0.90	NS	2.04±	1.97±	NS
Therapy							
ACEI/ARB (*n*, %)	65 (82)	52 (82)	50 (82)	NS	71 (78)	29 (88)	0.021
BB (*n*, %)	22 (28)	18 (28)	13 (21)	NS	15 (16)	16 (48)	<0.001
Statin (*n*, %)	26 (33)	18 (28)	18 (46)	NS	22 (24)	13 (39)	0.027
CCB (*n*, %)	22 (28)	12 (19)	18 (29)	NS	18 (20)	12 (36)	0.015

BMI: body mass index; eGFR: estimated glomerular filtration rate; ACEI: angiotensin-converting enzyme inhibitor; ARB: angiotensin receptor blocker; BB: beta-blocker; CCB: calcium channel blocker; LVEF: left ventricular ejection fraction; LVMI: left ventricular mass index; LVM: left ventricular mass; DD: diastolic dysfunction; Hb: hemoglobin; AU: urine albuminuria; UA: uric acid; HDL: high-density lipoprotein; TG: triglyceride. NS: not significant.

**Table 2:** Correlations.

	PAR		PLR		PLT	
	r	*p*	r	*p*	r	*p*
Gender	−0.273	0.001	0.031	0.367	−0.201	0.013
Age	−0.007	0.468	−0.029	0.377	−0.128	0.079
Dyslipidemia	0.073	0.209	0.005	0.477	0.084	0.176
Obesity	−0.024	0.397	−0.079	0.192	−0.077	0.198
HT	0.068	0.227	0.077	0.198	0.073	0.211
DM	−0.064	0.239	−0.097	0.141	−0.131	0.074
eGFR (ml/min)	0.056	0.266	0.143	0.057	0.158	0.040
AU (mg/L)	0.048	0.296	−0.165	0.033	0.038	0.336
M	0.081	0.205	0.056	0.283	0.084	0.194
E	0.033	0.367	−0.041	0.334	0.045	0.319
S	0.161	0.047	0.001	0.497	0.087	0.185
T	−0.016	0.435	0.019	0.423	−0.058	0.274
C	0.053	0.292	0.069	0.237	0.061	0.264
LVH	0.003	0.486	−0.178	0.025	−0.130	0.077

PAR: platelet-to-albumin ratio; PLR: platelet-to-lymphocyte ratio; PLT: platelet; HT: hypertension; DM: diabetes mellitus; eGFR: estimated glomerular filtration rate; AU: albuminuria; M: mesangial hypercellularity in histology; E: endocapillary hypercellularity; S: segmental glomerulosclerosis; T: tubular atrophy/interstitial fibrosis; C: cellular or fibrocellular crescents; LVH: left ventricular hypertrophy.

**Table 3:** Uni-and multivariate regression analysis of PAR, PLR, and PLT.

	Univariate Analysis					Multivariate Analysis						
PAR	B	Std. Errors	Beta	t	*p*	B	Std. Errors	Beta	t	*p*	95.0% CI for B Lower	95.0% CI for B Upper
Gender	−1.098	0.348	−0.273	−3.153	0.002	−1.264	0.418	−0.315	−3.025	0.003	−2.094	−0.434
Age	−0.001	0.013	−0.007	−0.080	0.937	0.005	0.017	0.033	0.296	0.768	−0.028	0.038
Dyslipidemia	0.278	0.342	0.073	0.812	0.418	0.351	0.395	0.092	0.888	0.377	−0.434	1.136
Obesity	−0.105	0.400	−0.024	−0.261	0.794	0.300	0.511	0.068	0.587	0.559	−0.716	1.316
HT	0.297	0.395	0.068	0.751	0.454	0.669	0.510	0.152	1.313	0.193	−0.344	1.683
DM	−0.285	0.400	−0.064	−0.712	0.478	−0.447	0.525	−0.101	−0.851	0.397	−1.490	0.596
eGFR	0.003	0.005	0.056	0.627	0.532	0.008	0.006	0.156	1.298	0.197	−0.004	0.021
AU	0.001	0.001	0.048	0.537	0.592	0.001	0.001	0.063	0.607	0.545	0.001	0.002
M	0.305	0.369	0.081	0.828	0.410	0.470	0.391	0.124	1.200	0.233	−0.308	1.247
E	0.655	1.921	0.033	0.341	0.734	0.845	1.979	0.043	0.427	0.670	−3.087	4.777
S	0.760	0.451	0.161	1.687	0.095	0.848	0.518	0.180	1.638	0.105	−0.180	1.876
T	−0.041	0.249	−0.016	−0.163	0.871	−0.070	0.291	−0.027	−0.240	0.811	−0.648	0.508
C	0.230	0.418	0.053	0.550	0.583	−0.028	0.459	−0.006	−0.061	0.951	−0.940	0.883
LVH	0.012	0.346	0.003	0.034	0.973	−0.115	0.453	−0.030	−0.253	0.801	−1.015	0.785
**PLR**												
Gender	4.241	12.486	0.031	0.340	0.735	6.407	1.524	0.046	0.441	0.660	−22.442	35.256
Age	−0.144	0.456	−0.029	−0.315	0.753	0.324	0.574	0.064	0.563	0.575	−0.817	1.464
Dyslipidemia	0.695	11.831	0.005	0.059	0.953	2.324	13,736	0.018	0.169	0.866	−24.961	29.608
Obesity	−12.019	13.772	−0.079	−0.873	0.385	−11.937	17.775	−0.078	−0.672	0.504	−47.244	23.370
HT	11.600	13.622	0.077	0.852	0.396	29.988	17.724	0.199	1.692	0.094	−5.218	65.195
DM	−14.860	13.750	−0.097	−1.081	0.282	−15.847	18.245	−0.104	−0.869	0.387	−52.088	20.394
eGFR	0.261	0.164	0.143	1.590	0.114	0.341	0.222	0.186	1.535	0.128	−0.100	0.782
AU	−0.017	0.009	−0.165	−1.850	0.067	−0.020	0.011	−0.195	−1.864	0.066	−0.041	0.001
M	7.295	12.696	0.056	0.575	0.567	10.273	13.604	0.079	0.755	0.452	−16.750	37.297
E	−28.315	65.985	0.041	−0.429	0.669	−56.341	68.790	−0.082	−0.819	0.415	−192.983	80.302
S	0.135	15.688	0.001	0.009	0.993	14.410	17.985	0.089	0.801	0.425	−21.315	50.134
T	1.671	8.540	0.019	0.196	0.845	12.890	10.112	0.146	1.275	0.206	−7.196	32.976
C	10.334	14.354	0.069	0.720	0.473	4.195	15.947	0.028	0.263	0.793	−27.481	35.871
LVH	−23.245	11.713	−0.178	−1.985	0.049	−27.749	15.746	−0.213	−1.762	0.081	−59.026	3.528
**PLT**												
Gender	−27.550	12.153	−0.201	−2.267	0.025	−35.340	14.137	−0.258	−2.500	0.014	−63.421	−7.259
Age	−0.639	0.449	−0.128	−1.422	0.157	−0.318	0.559	−0.063	−0.568	0.571	−1.428	0.793
Dyslipidemia	10.936	11.708	0.084	0.934	0.352	14.666	13.370	0.113	1.097	0.276	−11.892	41.223
Obesity	−11.645	13.680	−0.077	−0.851	0.396	2.471	17.301	0.016	0.143	0.887	−31.896	36.838
HT	10.901	13.533	0.073	0.806	0.422	35.171	17.252	0.235	2.039	0.044	0.902	69.441
DM	−19.848	13.602	−0.131	−1.459	0.147	−19.807	17.759	−0.131	−1.115	0.268	−55.083	15.469
eGFR	0.287	0.163	0.158	1.765	0.080	0.398	0.216	0.219	1.843	0.069	−0.031	0.827
AU	0.004	0.009	0.038	0.425	0.672	0.007	0.010	0.065	0.638	0.525	−0.014	0.027
M	10.929	12.584	0.084	0.869	0.387	15.123	13.242	0.117	1.142	0.256	−11.180	41.427
E	30.863	65.520	0.045	0.471	0.639	9.932	66.958	0.015	0.148	0.882	−123.073	142.936
S	13.965	15.521	0.087	0.900	0.370	18.098	17.506	0.112	1.034	0.304	−16.676	52.871
T	−5.094	8.469	−0.058	−0.601	0.549	0.813	9.843	0.009	0.083	0.934	−18.738	20.364
C	9.038	14.263	0.061	0.634	0.528	−0.481	15.522	−0.003	−0.031	0.975	−31.313	30.352
LVH	−16.848	11.722	−0.130	−1.437	0.153	−15.306	15.327	−0.118	−0.999	0.321	−45.751	15.138

PAR: platelet-to-albumin ratio; PLR: platelet-to-lymphocyte ratio; PLT: platelet; HT: hypertension; DM: diabetes mellitus; eGFR: estimated glomerular filtration rate; AU: albuminuria; M: mesangial hypercellularity in histology; E: endocapillary hypercellularity; S: segmental glomerulosclerosis; T: tubular atrophy/interstitial fibrosis; C: cellular or fibrocellular crescents; LVH: left ventricular hypertrophy.

**Table 4:** Multivariate Cox regression analysis with the primary combined, secondary renal, and cardiovascular endpoints.

Primary, Combined Endpoints	B	SE	Wald	df	*p*	Exp(B)	95.0% CI for Exp(B) Lower	95.0% CI for Exp(B) Upper
PLR	0.009	0.004	4.903	1	0.027	1.009	1.001	1.017
PAR	0.734	0.465	2.489	1	0.115	2.084	0.837	5.188
PLT	−0.019	0.013	2.048	1	0.152	0.981	0.957	1.007
Gender	−2.021	0.778	6.740	1	0.009	0.133	0.029	0.609
Age	0.035	0.023	2.277	1	0.131	1.035	0.990	1.083
Dyslipidemia	1.186	0.564	4.421	1	0.036	3.273	1.084	9.885
Obesity	0.523	0.507	1.067	1	0.302	1.688	0.625	4.556
HT	−1.262	1.171	1.162	1	0.281	0.283	0.029	2.810
DM	−1.354	0.589	5.280	1	0.022	0.258	0.081	0.819
eGFR (mL/min/1.73 m^2^)	−0.015	0.010	2.556	1	0.110	0.985	0.966	1.003
AU (mg/L)	0.001	0.001	1.567	1	<0.001	1.001	1.001	1.002
M	0.509	0.527	0.933	1	0.334	1.663	0.593	4.668
E	9.206	528.463	0.001	1	0.986	9954.897	0.001	6781.987
S	0.457	0.646	0.500	1	0.479	1.579	0.445	5.604
T	0.660	0.657	1.009	1	0.315	1.936	0.534	7.022
C	−0.450	0.535	0.705	1	0.401	0.638	0.223	1.821
LVH	−0.892	0.592	2.273	1	0.132	0.410	0.129	1.307
Secondary renal endpoints								
PLR	0.003	0.006	0.269	1	0.604	1.003	0.991	1.015
PAR	0.337	0.629	0.288	1	0.592	1.401	0.409	4.804
Tct	−0.016	0.018	0.768	1	0.381	0.984	0.951	1.020
Gender	0.140	0.675	0.043	1	0.836	1.150	0.306	4.316
Age	−0.008	0.027	0.079	1	0.778	0.992	0.941	1.047
Dyslipidemia	0.277	0.676	0.167	1	0.683	1.319	0.350	4.965
Obesity	0.458	0.608	0.569	1	0.451	1.581	0.481	5.204
HT	−2.379	1.375	2.991	1	0.084	0.093	0.006	1.373
DM	−0.332	0.769	0.187	1	0.666	0.717	0.159	3.236
eGFR (mL/min/1.73 m^2^)	−0.011	0.011	1.011	1	0.315	0.989	0.967	1.011
AU (mg/L)	0.002	0.001	15.021	1	0.001	1.002	1.001	1.003
M	0.829	0.718	1.331	1	0.249	2.290	0.560	9.359
E	9.946	700.758	0.001	1	0.989	20,870.393	0.001	12,678.798
S	−0.512	0.763	0.450	1	0.502	0.599	0.134	2.676
T	−0.145	0.839	0.030	1	0.863	0.865	0.167	4.483
C	0.956	0.812	1.383	1	0.240	2.600	0.529	12.778
LVH	−1.880	0.896	4.401	1	0.036	0.153	0.026	0.884
Secondary cardiovascular endpoints								
PLR	0.007	0.005	1.773	1	0.183	1.007	0.997	1.018
PAR	0.485	0.808	0.360	1	0.548	1.624	0.333	7.911
PLT	0.013	0.026	0.271	1	0.602	1.014	0.964	1.066
Gender	−3.753	1.482	6.412	1	0.011	0.023	0.001	0.428
Age	0.137	0.064	4.595	1	0.032	1.147	1.012	1.300
Dyslipidemia	1.932	1.151	2.816	1	0.093	6.902	0.723	65.888
Obesity	1.271	1.294	0.965	1	0.326	3.563	0.282	44.995
HT	−12.897	262.853	0.002	1	0.961	0.001	0.001	0.0001
DM	−2.279	1.029	4.905	1	0.027	0.102	0.014	0.769
eGFR (mL/min/1.73 m^2^)	−0.039	0.028	2.009	1	0.156	0.962	0.911	1.015
AU (mg/L)	0.001	0.001	0.234	1	0.629	1.000	0.999	1.002
M	1.180	1.175	1.010	1	0.315	3.256	0.326	32.551
E	8.193	1722.647	0.001	1	0.996	3615.569	0.001	2356.432
S	3.838	2.102	3.332	1	0.068	46.413	0.754	2858.117
T	1.857	1.825	1.035	1	0.309	6.407	0.179	229.266
C	−0.912	1.041	0.766	1	0.381	0.402	0.052	3.094
LVH	−1.799	1.252	2.063	1	0.151	0.165	0.014	1.926

PAR: platelet-to-albumin ratio; PLR: platelet-to-lymphocyte ratio; PLT: platelet; HT: hypertension; DM: diabetes mellitus; eGFR: estimated glomerular filtration rate; AU: albuminuria; M: mesangial hypercellularity in histology; E: endocapillary hypercellularity; S: segmental glomerulosclerosis; T: tubular atrophy/interstitial fibrosis; C: cellular or fibrocellular crescents; LVH: left ventricular hypertrophy.

## Data Availability

The data underlying this article cannot be shared publicly due to Hungarian regulations and the privacy of individuals who participated in the study. The data could be shared on reasonable request to the corresponding author if accepted by the Regional Committee for Medical and Health Research Ethics and local Data Protection Officials.
